# A computational model to explore how temporal stimulation patterns affect synapse plasticity

**DOI:** 10.1371/journal.pone.0275059

**Published:** 2022-09-23

**Authors:** Ryota Amano, Mitsuyuki Nakao, Kazumichi Matsumiya, Fumikazu Miwakeichi

**Affiliations:** 1 Graduate School of Information Sciences, Tohoku University, Sendai, Japan; 2 Department of Statistical Modeling, The Institute of Statistical Mathematics, Tachikawa-Shi, Japan; Georgia State University, UNITED STATES

## Abstract

Plasticity-related proteins (PRPs), which are synthesized in a synapse activation-dependent manner, are shared by multiple synapses to a limited spatial extent for a specific period. In addition, stimulated synapses can utilize shared PRPs through synaptic tagging and capture (STC). In particular, the phenomenon by which short-lived early long-term potentiation is transformed into long-lived late long-term potentiation using shared PRPs is called “late-associativity,” which is the underlying principle of “cluster plasticity.” We hypothesized that the competitive capture of PRPs by multiple synapses modulates late-associativity and affects the fate of each synapse in terms of whether it is integrated into a synapse cluster. We tested our hypothesis by developing a computational model to simulate STC, late-associativity, and the competitive capture of PRPs. The experimental results obtained using the model revealed that the number of competing synapses, timing of stimulation to each synapse, and basal PRP level in the dendritic compartment altered the effective temporal window of STC and influenced the conditions under which late-associativity occurs. Furthermore, it is suggested that the competitive capture of PRPs results in the selection of synapses to be integrated into a synapse cluster via late-associativity.

## Introduction

Long-term potentiation (LTP) is a widespread phenomenon found at most excitatory synapses and is essential in experience-dependent changes in brain function [1]. Moreover, such synaptic plasticity could be a fundamental mechanism in memory formation in the brain [2]. N-methyl-D-aspartate receptor (NMDAR)-dependent LTP can be classified into two types: early LTP (e-LTP), which has a short maintenance period independent of the synthesis of novel plasticity-related proteins (PRPs), and late LTP (l-LTP) with long maintenance period, which requires the synthesis of PRPs. Furthermore, each stage splits into three events: induction, maintenance, and expression [3,4]. Active cAMP response element-binding protein (CREB) at least partially triggers the synthesis of PRPs and is essential for forming hippocampal-dependent long-term memory [5]. The mechanism by which PRPs are specifically utilized at stimulated synapses is described by synaptic tagging and capture (STC) theory [6]. STC is a simple model in which synaptic tag are expressed at stimulated synapses in a protein synthesis-independent manner, and synapses with synaptic tag can specifically take up PRPs. “Late-associativity,” in which e-LTP-induced synapses capture proteins from the shared pool and translocate them to l-LTP, has also been reported [7]. “Late-associativity” is the principle underlying the “clustered plasticity” model, which predicts that accelerated local synthesis of proteins and STC will lead to synapse clustering [8]. Clustered plasticity enhances the efficiency of long-term memory formation, recovery, and capacity in individual neurons [9]. Temporal ordering of memory may benefit from the interaction between global plasticity regulation via CREB activity in the cell body and local plasticity regulation such as clustered plasticity [10,11]. Clustered plasticity may also be influenced by the spatial distribution of synapses [12], NMDARs [13], turnover of spines before learning [14], and activation of tropomyosin receptor kinase B (TrkB) by a brain-derived neurotrophic factor (BDNF) [15]. Also, the late-associativity underlying clustered plasticity may be affected by locally synthesized PRPs [16,17] and their competitive capture, but its effects have only been noted in limited studies. At the very least, there is a temporal window in which STC function effectively [12], and competition for PRP capture occurs between synapses when proteins are depleted [18,19]. Because the number of synapses constituting an approximately 10-μm cluster is at most 10, there may be a mechanism to select synapses for integration into a cluster [20]. In addition, the conditions under which l-LTP requires new protein synthesis are limited [21,22], and the temporal window of STC might vary with the basal PRP level in the dendritic compartment.

We were interested in the mechanism by which synapses that are potentiated by late-associativity are selected during synapse cluster formation. Specifically, our study analyzed the effects of the number of competing synapses, timing of stimulation to each synapse, and basal PRP level in the dendritic compartment on late-associativity. These factors appeared to influence the competitive capture of PRPs. Therefore, we developed a simple computational model to simulate the classic STC experiments, late-associativity, and competitive capture of PRPs [6,7]. This model assumes a spatially uniform dendritic compartment for PRPs, and the basal PRP level within the compartment is adjustable. First, we confirmed that our model could simulate the late-associativity and STC asymmetric temporal windows reported in classical experiments [7,12]. Next, we analyzed the effects of the number of competing synapses, timing of stimulation to each synapse, and basal PRP level in the dendritic compartment on late-associativity. The results suggested that late-associativity has a pronounced time window when the basal PRP level is in the appropriate range and displays different enhancement tendencies in multiple synapses depending on the stimulation pattern.

## Materials and methods

### Model structure and simulation environment

**Fig 1:**
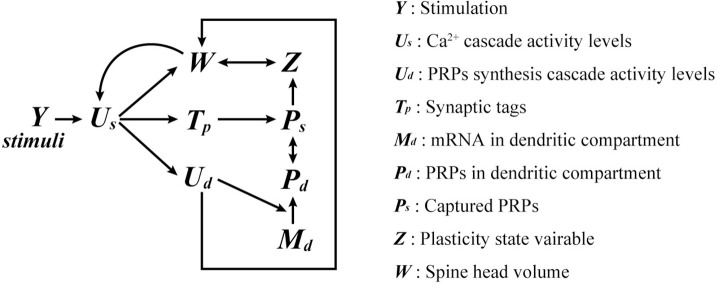
A schematic diagram of our model. The relationships between the variables formulated in Eqs (1)–(8). Arrows mean that the variable following the endpoint of the arrow depends on the variable preceding the starting point.

The sharing and competitive capture of *de novo* PRPs can occur in a restricted space [12,18,19]. We targeted a 10-μm-long dendritic compartment for modeling, which we believe ensures spatial uniformity among PRPs [12]. The spine density of hippocampal pyramidal cells depends on age, region, and distance from the soma, and the number of spines placed in the compartment was assumed to be 2.0/μm in the model [23,24]. Thus, a maximum of 20 spines were placed in our model. Fig 1 presents a diagram of the model. Table 1 presents the model parameters used in the simulations. In the simulations, we labeled synapses that induced l-LTP as L1 and those that induced e-LTP as En, where n = 1, 2,…, 19. We denoted the timing of induction protocols for synapse E1 as *s*_*E1*_. The response of each synapse to protocols was quantified as the spine head volume 180 min after stimulation.

**Table 1:** Model parameters.

Parameter	Standard Value	Parameter	Standard Value
$k_{U_{s}}$[/sec]	1.0×10^3^	$K_{P_{s}}$	0.1
$\beta_{U_{s}}$[/sec]	0.1	$h_{P_{s}}$	1
$k_{U_{d}}$[/sec]	0.1	$\beta_{P_{s}}$[/sec]	5.0×10^−3^
$\alpha_{U_{d}}$	1.0	$k_{T_{P}}$[/sec]	0.2
$\beta_{U_{d}}$[/sec]	1.0×10^−3^	$K_{T_{P}}$	5.0×10^−2^
$K_{U_{d}}$	0.8	$h_{T_{P}}$	8
$h_{U_{d}}$	8	$\tau_{T_{P}}$[sec]	2.0×10^4^
$\tau_{M_{d}}$[sec]	1.0×10^4^	$\mu_{T_{P}}$	0.1
$\tau_{P_{d}}$[sec]	2.0×10^2^	*k*_*Z*_[/sec]	5.0×10^−4^
$\mu_{M_{d}}$	1.0	*K*_*Z*_	6.0×10^−2^
$\mu_{P_{d}}$	1.0×10^−3^	*h*_*Z*_	8
$k_{P_{d}}$[/sec]	5.0×10^2^	*k*_*W*_[/sec]	3.0×10^−3^
$K_{P_{d}}$	0.2	*K*_*W*_	5.0×10^−2^
$h_{P_{d}}$	8	*h*_*W*_	8
$k_{P_{s}}$[/sec]	3.0×10^−3^	*τ*_*W*_[sec]	3.3×10^2^

Parameter standard values used throughout all simulations.

### e-LTP/l-LTP–inducing stimulation protocols, Ca^2+^ cascades, and PRP synthesis cascades

e-LTP induction requires NMDA receptor-mediated Ca^2+^ influx into synapses and the activation of Ca^2+^ cascades. We defined the activity level of the stimulus-dependent Ca^2+^ cascade as *U*_*s*_ (Eq 1) and assumed that the synaptic skeleton expands and synaptic tag synthesis is accelerated when the *U*_*s*_ level exceeds the threshold. Conversely, the l-LTP–inducing stimulation protocol was distinguished from the e-LTP–inducing stimulation protocol because its protocol induced *de novo* protein synthesis. We defined the activity level of the PRP synthesis cascade in dendrites as *U*_*d*_ (Eq 2) and hypothesized that the synthesis of *de novo* PRPs would be accelerated when the *U*_*d*_ level exceeds the threshold. As the stimulation protocol, we adopted glutamine uncaging and adjusted the model parameters so that e-LTP was induced by 30 pulses of stimulation in units of 5 ms at 0.5 Hz [12]. In addition, l-LTP was induced using the PKA pathway agonist forskolin (FSK) or the D1R agonist SKF38393 (SKF) in combination with the e-LTP–inducing stimulation protocol. FSK and FKS were expected to promote the synthesis of novel proteins in the dendrite compartment [12]. Therefore, in our model, the effects of FSK and FKS are reflected by the coefficient $\alpha_{U_{d}}$ in Eq 2 as follows:

$$\frac{dU_{s}^{\left[n\right]}}{dt}=k_{U_{s}}W^{\left[n\right]}Y^{\left[n\right]}\left(1-U_{s}^{\left[n\right]}\right)-\beta_{U_{s}}U_{s}^{\left[n\right]}$$ (1)

$$\frac{dU_{d}}{dt}=k_{U_{d}}\Theta \left[\sum_{n=1}^{N}\alpha_{U_{d}}\beta_{U_{s}}U_{s}^{\left[n\right]},K_{U_{d}},h_{U_{d}}\right](1-U_{d})-\beta_{U_{d}}U_{d}$$ (2)

where *X*^[*n*]^ denotes a variable *X* defined in the nth spine, *U*_*s*_^[*n*]^ and *U*_*d*_ are the normalized activity levels of the stimulus-dependent Ca^2+^ cascades in spines and of the PRP synthesis cascades in dendrites (*U*_*s*_^[*n*]^, *U*_*d*_ = 0 to 1), respectively, *W*^*[n]*^ is the spine head volume (μm^3^), and *Y*^*[n]*^ is the intensity of the stimulus (*Y*^*[n]*^ = 0–1). The transition speeds of *U*_*s*_^[*n*]^ from 0 to 1 and 1 to 0 were set to $k_{U_{s}}=1.0\times 10^{3}$ s^−1^ and $\beta_{U_{s}}=0.1$ s^−1^, respectively. Similarly, the transition velocities of *U*_*d*_ were set to $k_{U_{d}}=0.1$ s^−1^ and $\beta_{U_{d}}=1.0\times 10^{-3}$ s^−1^. In the l-LTP–inducing stimulation protocol, the standard value of $\alpha_{U_{d}}$ was multiplied by 100. $\Theta [X,K,h]=\frac{X^{h}}{X^{h}+K^{h}}$ denoted the Hill equation used in biochemical reactions. As presented in Eq 2, the PRP synthesis cascades were assumed to be activated by the Ca^2+^ cascade activity level of each spine. Conversely, activating the PRP synthesis cascade could promote synaptic plasticity [12]. We incorporated this feedback effect into the dynamics of the spine head volume as described in the Structural plasticity subsection.

### Protein synthesis

The specific location of local PRP synthesis in dendrites is unknown [25]. If local PRPs are synthesized in the spine, then the spine may be able to control its structural plasticity in a stimulus-specific manner. However, PRP synthesis could occur near the spine neck and dendrite shaft intersection [17]. Moreover, LTP rearranges polyribosomes and induces the accumulation of PRPs at the base of the spine for at least 2 h after stimulation [26,27]. In this case, PRP synthesis is not completely synapse-specific. The presence of late-associativity also suggests that there is a shared pool of PRPs in the dendrites. Therefore, we hypothesized that the synthesis of novel PRPs would occur in the dendrite compartment and incorporated PRPs and their source mRNAs into the model as a single variable. The dynamics of mRNA and PRPs were determined as follows:

$$\frac{dM_{d}}{dt}=-\frac{\left(M_{d}-\mu_{M_{d}}\right)}{\tau_{M_{d}}}-k_{P_{d}}\Theta [U_{d},K_{P_{d}},h_{P_{d}}]M_{d}$$ (3)

$$\frac{dP_{d}}{dt}=-\frac{\left(P_{d}-\mu_{P_{d}}\right)}{\tau_{P_{d}}}+k_{P_{d}}\Theta [U_{d},K_{P_{d}},h_{P_{d}}]M_{d}-\sum_{n=1}^{N}k_{P_{s}}\Theta [P_{d}T_{P}^{\left[n\right]},K_{P_{s}},h_{P_{s}}]+\sum_{n=1}^{N}\beta_{P_{s}}P_{s}^{\left[n\right]}$$ (4)

where *P*_*d*_ and *M*_*d*_ are the scaled concentrations of PRPs and their synthesis source mRNAs in the dendrites, respectively (0≤*P*_*d*_, *M*_*d*_). In Eq 3, we set the basal mRNA level in the dendrite compartment to $\mu_{M_{d}}=1.0$, and the time constant was set to $\tau_{M_{d}}=1.0\times 10^{4}$ s. For Eq 4, we assumed that the *P*_*d*_ synthesis rate exhibits nonlinearity that increases sharply near $U_{d}=K_{P_{d}}=0.2$. The basal PRP level $\mu_{P_{d}}$ in the dendrite compartment is a hyperparameter that can be changed, and the standard value is 1.0×10^−3^. The time constant of *P*_*d*_ was set to $\tau_{P_{d}}=2.0\times 10^{2}$ s. We hypothesized that each spine would capture PRPs by the STC method, but some captured PRPs would be fed back to the dendrites.

### STC

STC is a simple model in which synaptic tags are expressed at stimulated synapses, and synapses can capture PRPs. At a minimum, synaptic tagging requires actin-mediated cytoskeletal rearrangement and spine structural plasticity, which depends on CaMKII activity [28]. e-LTP expression and tagging are often observed simultaneously, but they can occur separately [29]. Moreover, synaptic tags are appropriate for referring to a state of plasticity rather than a single molecule [30]. We formulated the dynamics of a synaptic tag corresponding to the PRPs defined in Eq (4) as follows:

$$\frac{dT_{P}^{\left[n\right]}}{dt}=k_{T_{P}}W^{\left[n\right]}\Theta \left[U_{s}^{\left[n\right]},K_{T_{P}},h_{T_{P}}\right]\left(1-T_{P}^{\left[n\right]}\right)-\frac{(T_{P}^{\left[n\right]}-\mu_{T_{P}})}{\tau_{T_{P}}}$$ (5)

where *T*_*p*_^*[n]*^ is the scaled intensity of the synaptic tag, and larger values indicated stronger force for capturing PRPs ($0\leq T_{P}^{\left[n\right]}\leq 1$). We assumed that an increase in synaptic tag intensity is proportional to the product of the spine head volume *W*^*[n]*^ and the rate constant $k_{T_{P}}=0.2$ and that it exhibits nonlinearity that increases sharply near $U_{s}^{\left[n\right]}=K_{T_{p}}$. In addition, the synaptic tag intensity decays at the time constant $\tau_{T_{P}}=0.1$ s and returns to the baseline $\mu_{T_{P}}=0.1$.

Subsequently, we determined the capture dynamics of PRPs using synaptic tags as follows:

$$\frac{dP_{s}}{dt}=k_{P_{s}}\Theta [P_{d}T_{P}^{\left[n\right]},K_{P_{s}},h_{P_{s}}]-\beta_{P_{s}}P_{s}^{\left[n\right]}-k_{Z}\Theta [P_{s}^{\left[n\right]},K_{Z},h_{Z}](W^{\left[n\right]}-Z^{\left[n\right]})$$ (6)

where $P_{s}^{\left[n\right]}$ is the scaled concentration of PRPs captured by the spine ($0\leq P_{s}^{\left[n\right]}$). The capture rate depends on the product of the dendrite PRP concentration and synaptic tag strength [31], and the maximum rate is $k_{P_{s}}=3.0\times 10^{-3}$ s^−1^. $\tau_{P_{s}}$ is the diffusion rate from the spine to the dendritic compartment $\beta_{P_{s}}=5.0\times 10^{-3}$ s^−1^. As described in the next section, we assumed that the PRPs trapped in the spine are consumed as the plasticity state variable *Z*^*[n]*^ increases.

### Synaptic plasticity state

e-LTP decays in approximately 3 h, but l-LTP remains stable for more than 3 h [1,22]. In l-LTP, the structures of postsynaptic density (PSD) and presynapses change, and these changes contribute to the stabilization of l-LTP [32,33]. We devised a bistable model in which synapses’ basal and l-LTP states are stable and introduced the state variable *Z*^*[n]*^ of synaptic plasticity into the model. Many computational models use such bistability models [34–37]. In our model, *Z*^*[n]*^ = 0 represents the ground state of synapses. We also hypothesized that the enhancement of *Z*^*[n]*^ follows the enhancement of the spine head volume *W*^*[n]*^, which corresponds to the delayed changes in PSD and presynaptic structure after the enhancement of the spine skeleton [32,33]. We additionally hypothesized that PRPs are needed to enhance *Z*^*[n]*^. This assumption is consistent with the need for PRPs in l-LTP. *Z*^*[n]*^ was calculated as follows:

$$\frac{dZ^{\left[n\right]}}{dt}=k_{Z}\Theta [P_{s}^{\left[n\right]},K_{Z},h_{Z}](W^{\left[n\right]}-Z^{\left[n\right]})$$ (7)

where the initial value of *Z*^*[n]*^ was set to 0.10. This state variable increased depending on the amount of captured PRPs $P_{s}^{\left[n\right]}$ and eventually approached *W*^*[n]*^. The maximum rate of increase is *k*_*Z*_ = 5.0×10^−4^ s^−1^. We also assumed that there was no *Z*^*[n]*^ attenuation, and we described the validation of this assumption in the Discussion section.

### Structural plasticity

The spine head volume transiently expands up to five times upon stimulation and decays in approximately 3 h if the plasticity state does not change to l-LTP. Transient volume expansion depends on actin remodeling caused by the activation of the Ca^2+^ cascade [38]. Conversely, when a weak stimulus that does not cause structural plasticity in synapses is given to synapses in the vicinity of an l-LTP–induced synapse, stimulated synapses induce l-LTP. In summary, the induction of l-LTP locally promotes the induction of LTP via subsequent stimulation, but its molecular mechanism is unknown [12]. We hypothesized that the activity level of the PRP synthesis cascade *U*_*d*_ in dendrites promotes the induction of LTP and incorporated this variable into the increasing term of *W*^*[n]*^. In addition, as mentioned in the previous section, l-LTP is considered a type of structural equilibrium stage because the enlargement of the spine head volume is maintained in cooperation with PSD and presynaptic structural changes [33]. Therefore, we multiplied the attenuation term of *W*^*[n]*^ by the different term of *W*^*[n]*^ and the plasticity state variable *Z*^*[n]*^. The dynamics of the spine head volume were calculated as follows:

$$\frac{dW^{\left[n\right]}}{dt}=k_{W}\Theta [U_{s}^{\left[n\right]},K_{W},h_{W}](1+U_{d})-\frac{W^{\left[n\right]}(W^{\left[n\right]}-Z^{\left[n\right]})}{\tau_{W}}$$ (8)

where *W*^*[n]*^ is the spine head volume (initial value was set at 0.10 μm^3^) and increased at the maximum velocity *k*_*W*_(1+*U*_*d*_) according to the value of *U*_*s*_^[*n*]^. In this case, *k*_*W*_ = 3.0×10^−3^ s. *W*^*[n]*^ decays asymptotically to the plasticity state variable *Z*^*[n]*^ at the time constant *τ*_*W*_ = 3.3×10^2^ s. Because we assumed that an increase in the spine head volume precedes that of the plasticity state variable, *W*^[*n*]^≥*Z*^[*n*]^ always holds true. Therefore, the attenuation term satisfies $-\frac{W^{\left[n\right]}(Z^{\left[n\right]}-W^{\left[n\right]})}{\tau_{W}}\leq 0$. In addition, as can be determined from Eqs (7) and (8), when *Z*^*[n]*^ = *W*^*[n]*^, *Z*^*[n]*^ and *W*^*[n]*^ are in equilibrium, which is the l-LTP state.

## Results

### STC and late-associativity

**Fig 2:**
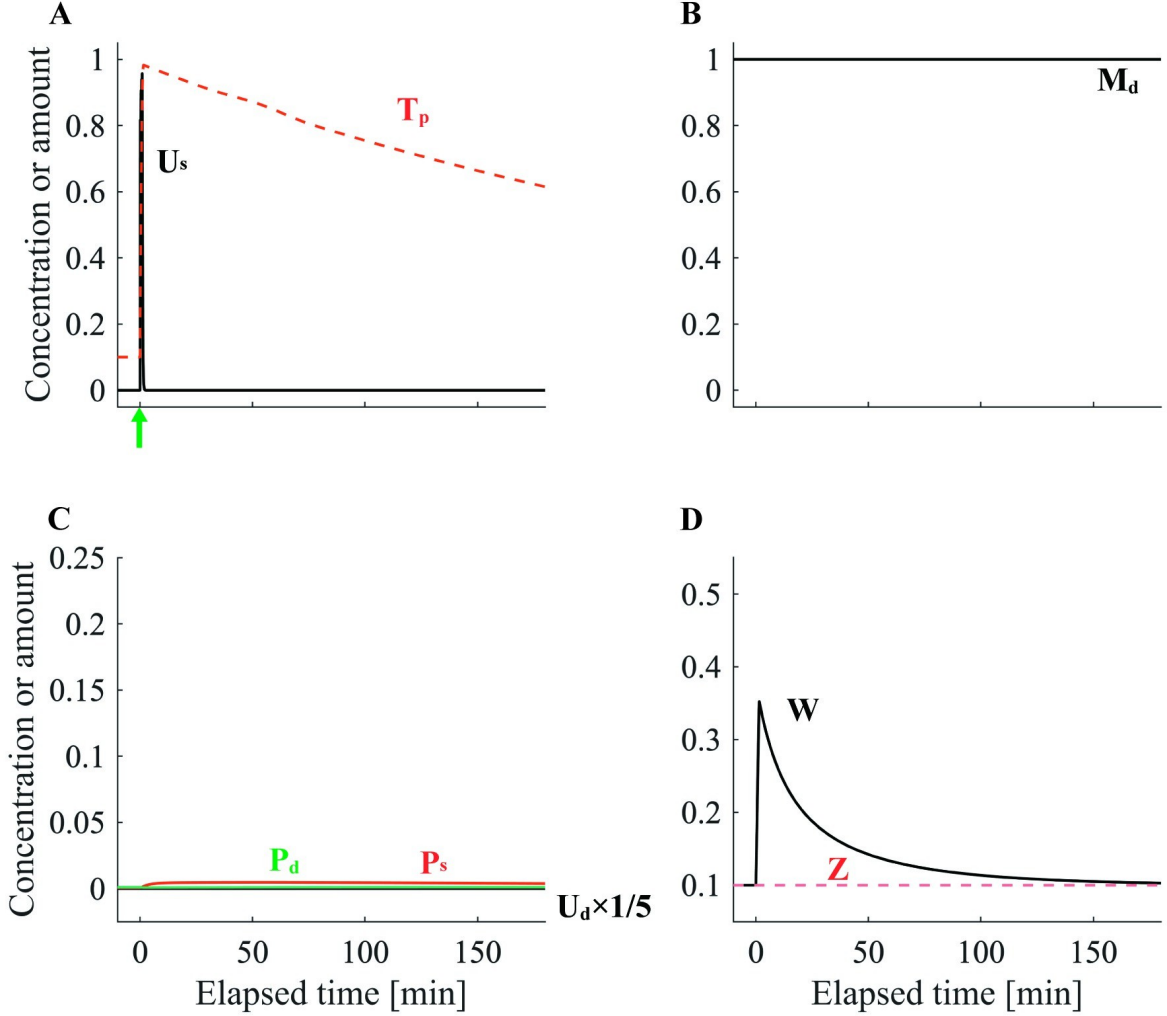
The dynamics of each variable in e-LTP. (A) The normalized activity level of stimuli-dependent Ca^2+^ cascades in the spine *U*_*s*_ (black). The scaled intensity of synaptic tag *T*_*p*_^*[n]*^ (red dashed line). The green arrow denotes the timing of the e-LTP–inducing stimulation protocol. (B) The scaled concentration of translation sources *M*_*d*_ (black). (C) The normalized activity level of PRP synthesis cascades in the dendritic compartment *U*_*d*_ (black). To improve visualization, *U*_*d*_ was scaled to 1/5. The scaled concentrations of synaptically trapped PRPs *P*_*s*_ (red) and PRPs in dendrites *P*_*d*_ (green) are presented. (D) The spine head volume *W* (black). State variable of synaptic plasticity *Z* (red dashed line).

**Fig 3:**
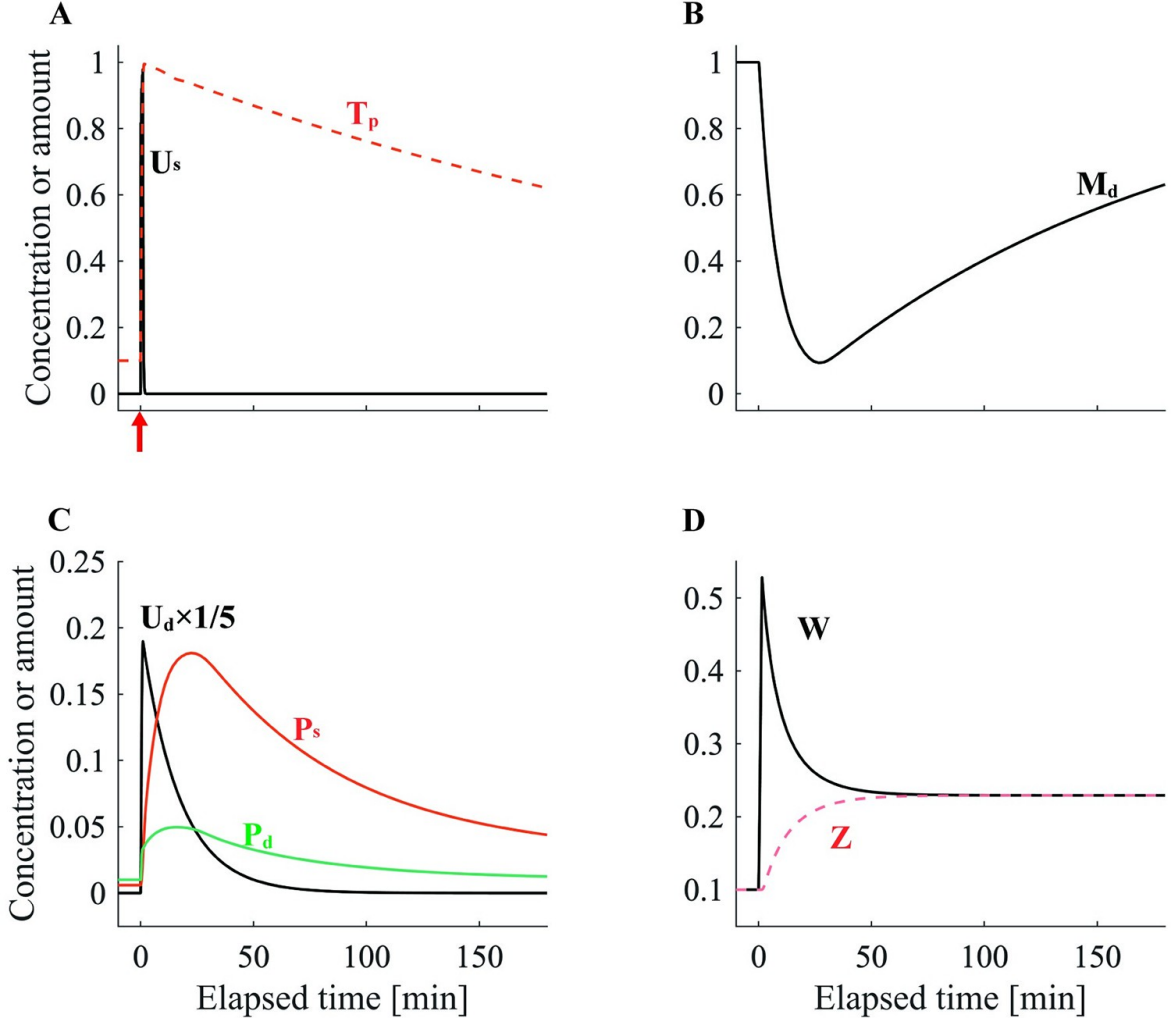
The dynamics of each variable in l-LTP. The variables illustrated in (A)–(D) are the same as those described in the caption of Fig 2. For (A), the red arrow indicates the timing of the l-LTP–inducing stimulation protocol.

We first simulated e-LTP and l-LTP and explored how the main parameters of our model were affected by each protocol. The e-LTP–inducing stimulation protocol enhanced the synaptic tag *T*_*p*_^*[n]*^ without triggering PRP synthesis. Therefore, the plasticity state variable *Z* was not enhanced, and the spine head volume *W* decayed completely within 180 min (Fig 2). In the l-LTP–inducing stimulation protocol, PRP synthesis caused *P*_*d*_ to rise and then slowly decay. Because *T*_*p*_^*[n]*^ was simultaneously enhanced, STC increased *P*_*s*_, which represents PRPs in the spine. Consequently, the plasticity state variable *Z*^*[n]*^ gradually increased with increasing *P*_*s*_ and asymptotically approached the spine head volume *W*^*[n]*^ (Fig 3). This means that the plasticity state has changed from e-LTP to l-LTP. Thus, this model simulates the qualitative properties of e-LTP and l-LTP [1].

**Fig 4:**
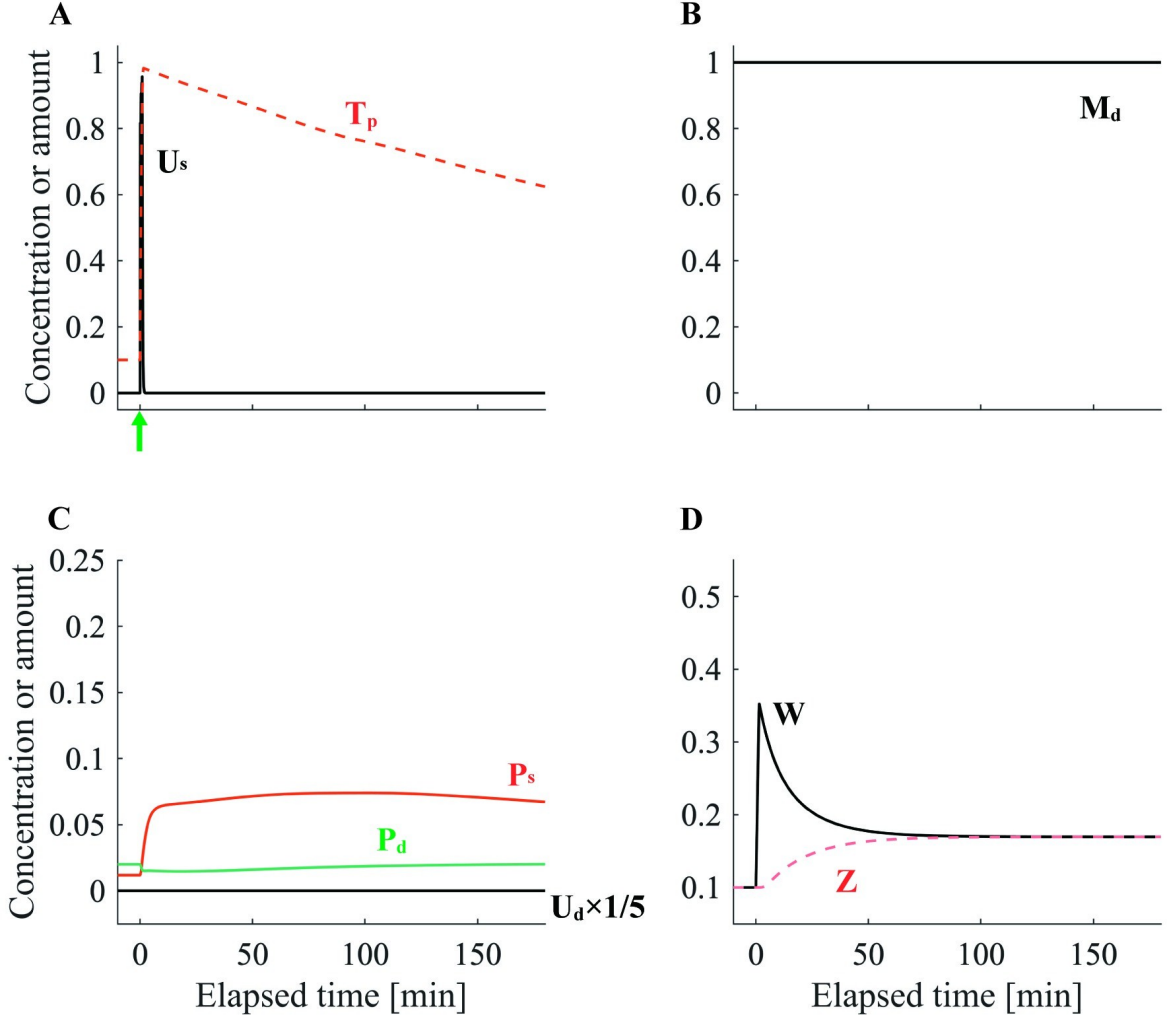
The dynamics of each variable with the e-LTP–inducing stimulation protocol under abundant PRP levels. The variables illustrated in (A)–(D) are the same as those described in the caption of Fig 2. For (A), the green arrow denotes the timing of the e-LTP–inducing stimulation protocol.

Conversely, it has been suggested that l-LTP does not require the synthesis of novel PRPs when the basal level of PRPs in the dendrite compartment is high [21,22]. Therefore, we set the basal PRP level $\mu_{P_{d}}$ to 50 times the standard value and performed the e-LTP–inducing stimulation protocol at synapse E1. Consequently, synapse E1 transmitted to l-LTP (Fig 4). By varying the basal PRP level, the model can reproduce experimental results with and without dependence on new protein synthesis [22].

**Fig 5:**
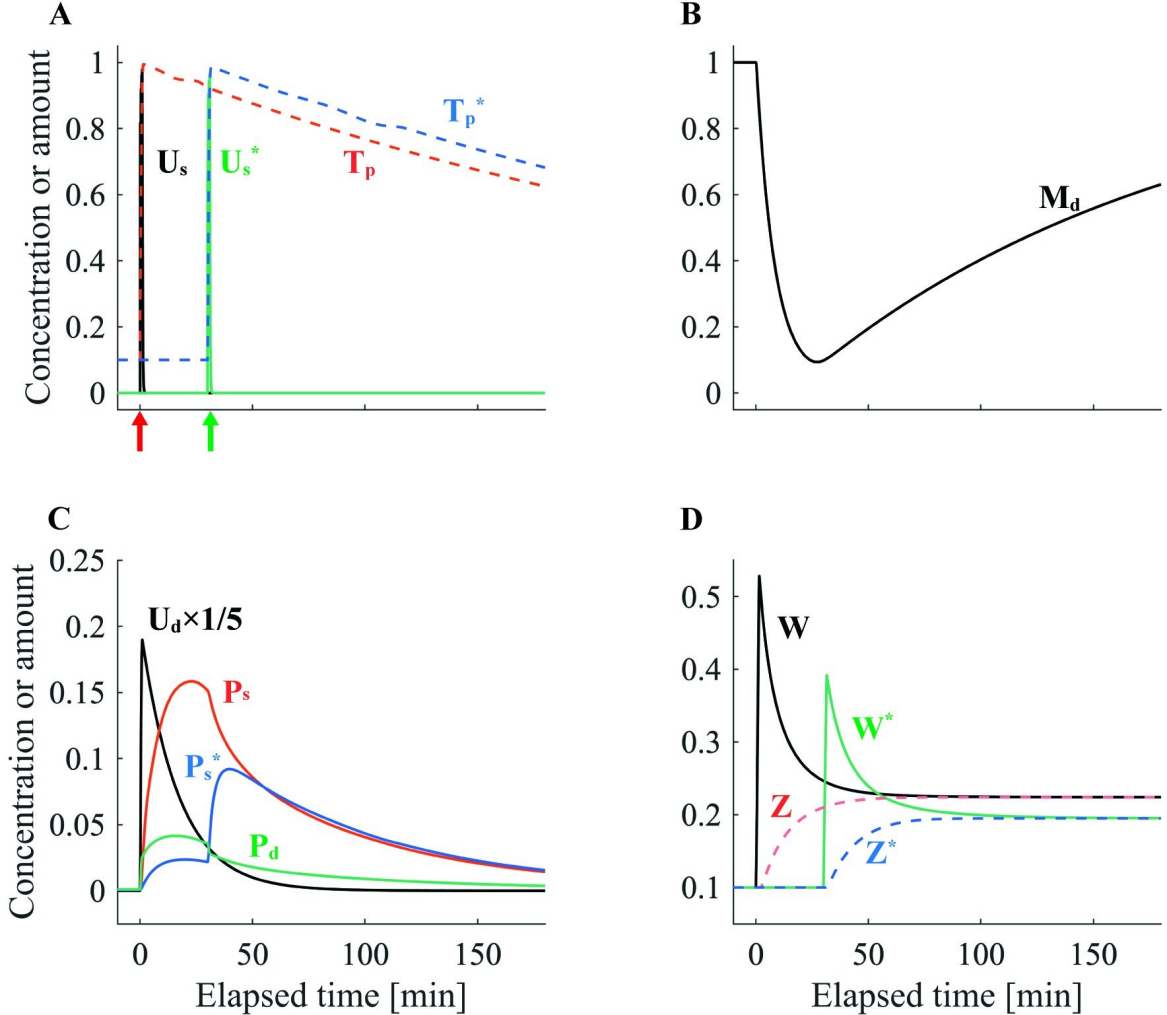
Simulation of late-associativity via STC. The variables illustrated in (A)–(D) are the same as those described in the caption of Fig 2. For (A), the green arrow denotes the timing of the e-LTP–inducing stimulation protocol, and the red arrow indicates that of the l-LTP–inducing stimulation protocol. The variables marked with asterisks are defined at the e-LTP–induced synapse.

Next, to confirm that our model can simulate late-associativity, we performed an e-LTP–inducing stimulation protocol at synapse E1 and an l-LTP–inducing stimulation protocol at synapse L1. As presented in Fig 5, synapse E1 also captured *de novo* PRPs, and the plasticity state variable *Z*^*[n]*^ was asymptotic to the spine head volume *W*^*[n]*^. Thus, our model can simulate the late-associativity reported in classical STC experiments [7].

### Temporal asymmetry of the STC window

**Fig 6:**
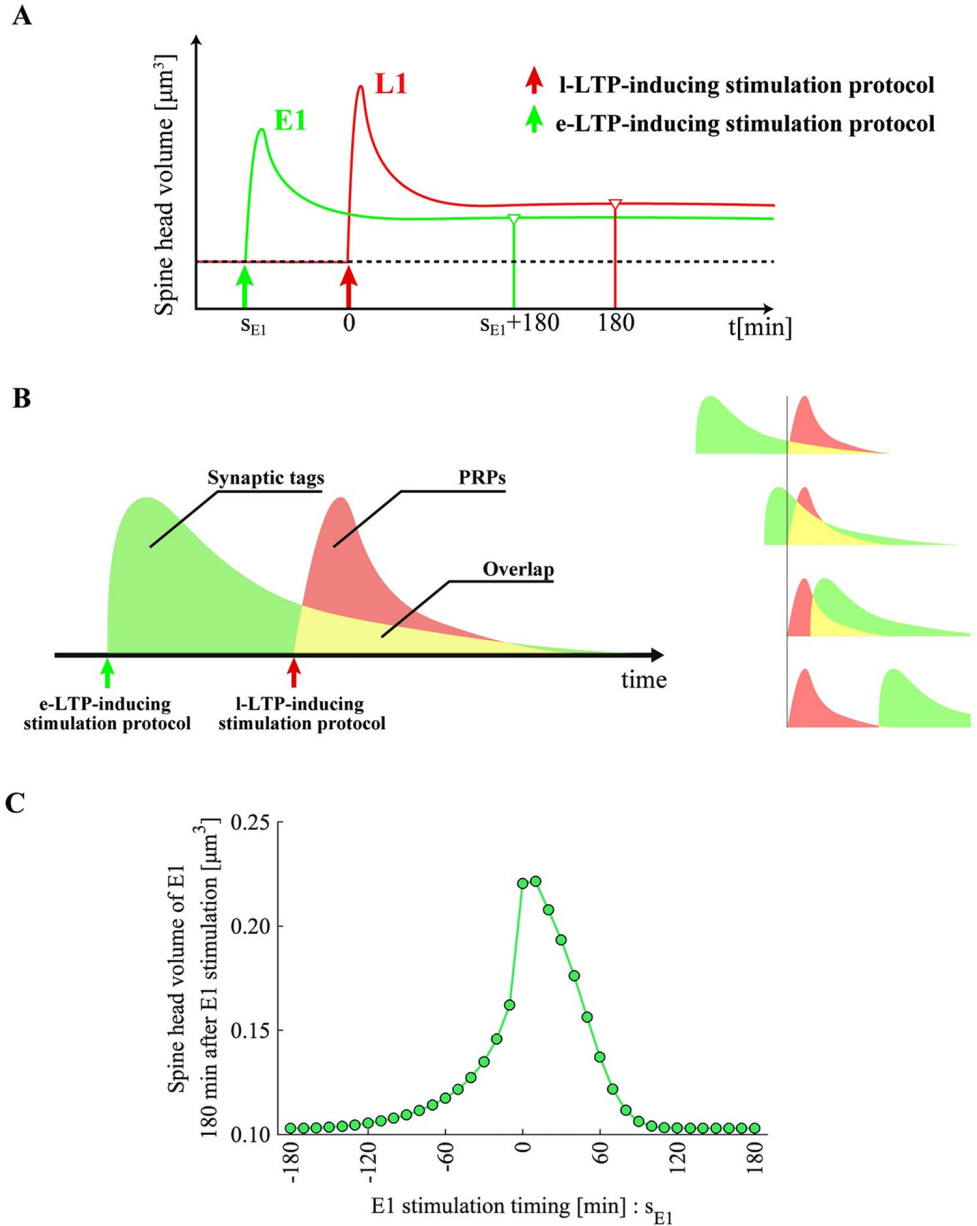
Temporal asymmetry of the STC window simulated by our model. (A) The schematic diagram of our experiment for simulating the temporal STC window. (B) Schematic illustration showing that the asymmetry of the STC temporal window is attributable to the difference in the time constants of the synaptic tag and newly synthesized PRPs. Yellow areas indicate the coexistence of synaptic tags and newly synthesized PRPs. The number of PRPs captured by synapses increases as the area increases. (C) A simulation result. Our model correctly simulated the asymmetry of the STC temporal window [12]. The horizontal axis denotes the relative timing of the e-LTP–inducing stimulation protocol at synapse E1 from the l-LTP–inducing stimulation protocol at synapse L1. The vertical axis denotes the spine head volume of E1 180 min after the e-LTP–inducing stimulation protocol to E1.

Next, we set the timing of applying the l-LTP–inducing stimulation protocol to synapse L1 as a reference (t = 0) and varied the timing of the e-LTP–inducing stimulation protocol to synapse E1 in the range of −180 to +180 min. Then, we quantified the response of synapses L1 and E1 to the induction protocol as the spine head volume 180 min after stimulation (Fig 6A). As Govindarajan et al. stated, the temporal window of STC depends on the time constant of synaptic tags and PRPs [12]. Therefore, we adjusted the time constants of *T*_*p*_^*[n]*^ and *P*_*d*_ so that the model traced the experimental results. Fig 6B shows a simple cartoon on the principle that differences in the time constants of the synaptic tags and PRPs produce asymmetry in the STC temporal window. In Fig 6C, the vertical axis denotes the spine head volume of synapse E1 180 min after stimulation, and the horizontal axis denotes the timing of the e-LTP induction protocol. Because we set the spine head volume in the basal state to 0.10 μm^3^, if the spine head volume 180 min after stimulation is also 0.10 μm^3^, this means that the transition from e-LTP to l-LTP has failed. On the contrary, if the value exceeds 0.10 μm^3^, then late-associativity has worked effectively. As presented in Fig 6C, when the e-LTP–inducing stimulation protocol precedes the l-LTP–inducing stimulation protocol, the period during which late-associativity is effective is relatively wide (approximately 120 min), and the spine head volume of E1 slowly decays in the negative direction of the horizontal axis. Conversely, when the e-LTP–inducing stimulation protocol follows the l-LTP–inducing stimulation protocol, the effective period of late-associativity is relatively narrow (approximately 90 min). Furthermore, the volume of synapse E1 decayed relatively steeply in the positive direction of the horizontal axis. Thus, our model correctly simulated the asymmetry of the STC temporal window, which corresponds to the effective temporal window of late-associativity [12].

### Simulation of competitive PRP capture

We performed two simulation experiments to analyze the effects of the number of competing synapses, timing of stimulation of each synapse, and basal PRP level in the dendritic compartment on late-associativity.

### Experiment I

**Fig 7:**
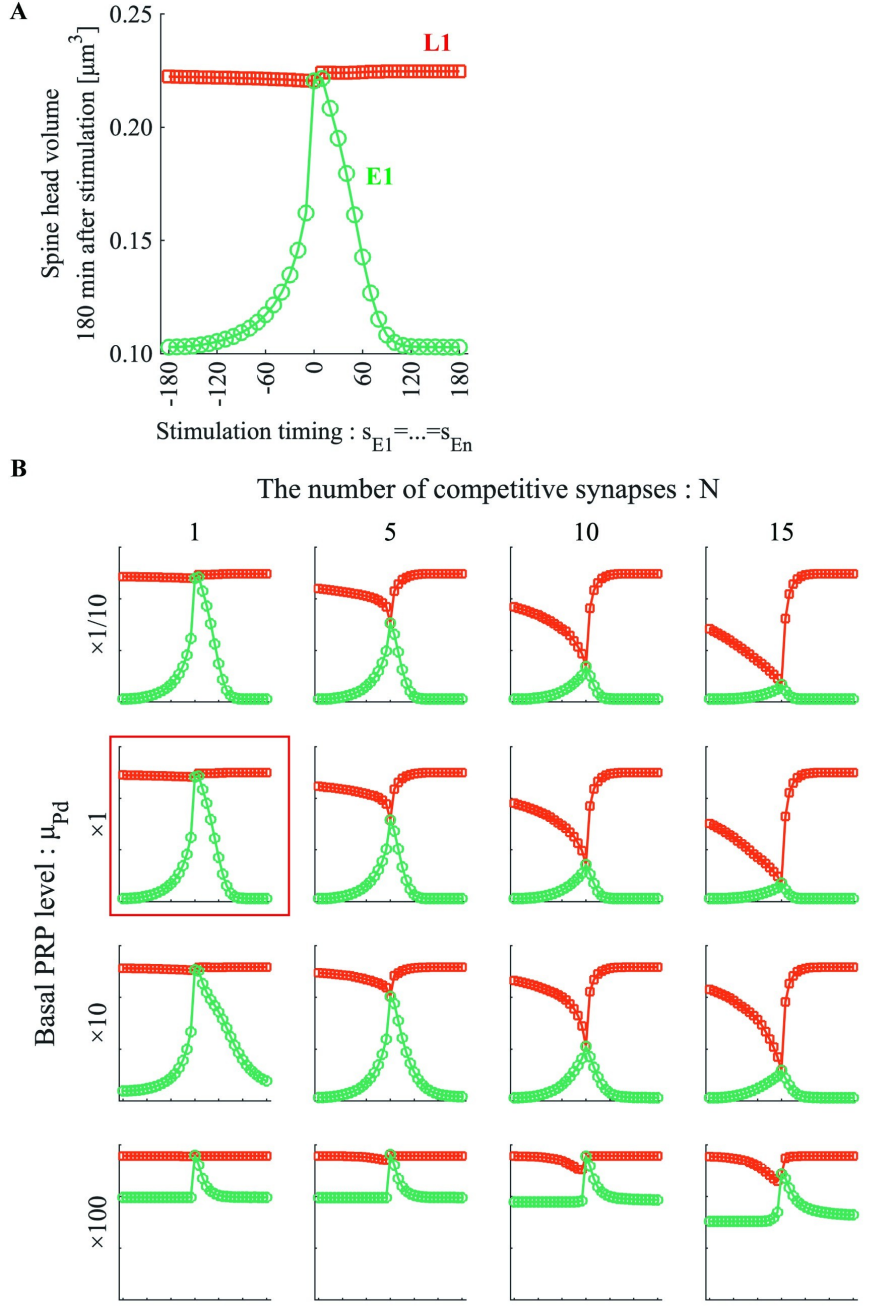
Influence of the number of competitive synapses and basal PRP level on the STC window. (A) Green circles and red squares indicate the volume of synapses E1 and L1, respectively, after potentiation. The horizontal axis is the execution timing of the e-LTP-inducing stimulation protocol relative to l-LTP–inducing stimulation. (B) The spine head volumes of E1 and L1 for different basal PRP levels and numbers of competitive synapses. In each panel, the scale of the vertical and horizontal axes is the same as that in (A). The panel with a red frame is the same as that in (A). “×10” in the basal PRP level means 10 times the standard value of $\mu_{P_{d}}$.

In experiment I, we applied the l-LTP–inducing stimulation protocol at synapse L1 and the e-LTP–inducing stimulation protocol at n synapses E1–En. Moreover, we completely synchronized the stimulation timings for synapses E1–En to reduce the degree of freedom of stimulus timing combinations (*s*_*E*1_ = *S*_*E*2_ = … = *S*_*En*_). Under this setting, we varied the number of synapses *N* to which the e-LTP–inducing stimulation protocol was applied and the basal PRP level $\mu_{P_{d}}$ in the dendritic compartment in several values (Fig 7).

In all panels of Fig 7, the vertical axis denotes the spine head volume 180 min after the e-LTP–inducing stimulation protocol, and the horizontal axis presents the stimulation timing *s*_*E*1_ min with the e-LTP–inducing stimulation protocol. The red square denotes synapse L1, and the green circle denotes synapse E1. Our model assumes that all spines are physically and biochemically equivalent, and thus, the amount of PRPs captured by synapses with synchronized stimulation timing is equal. Therefore, because the time courses of the model variables of synapses E1–En are equal, only the volume of synapse E1 is presented in Fig 7. In Fig 7B, the panel with the red frame is the same as that presented in Fig 7A. As presented in Fig 7B, the E1–En and L1 volumes decreased as the number of competing synapses *N* increased. When the basal PRP level in the dendritic compartment is high, the spine head volume of each synapse is large after potentiation, even if the number of competing synapses is large. In addition, regarding the STC temporal window, asymmetry is noticeable when the number of competing synapses is low and the basal PRP level is low. As the number of competing synapses increased, the maximum value of the temporal window decreased while maintaining its shape, but increases in the basal PRP level deformed the temporal window.

### Experiment II

**Fig 8:**
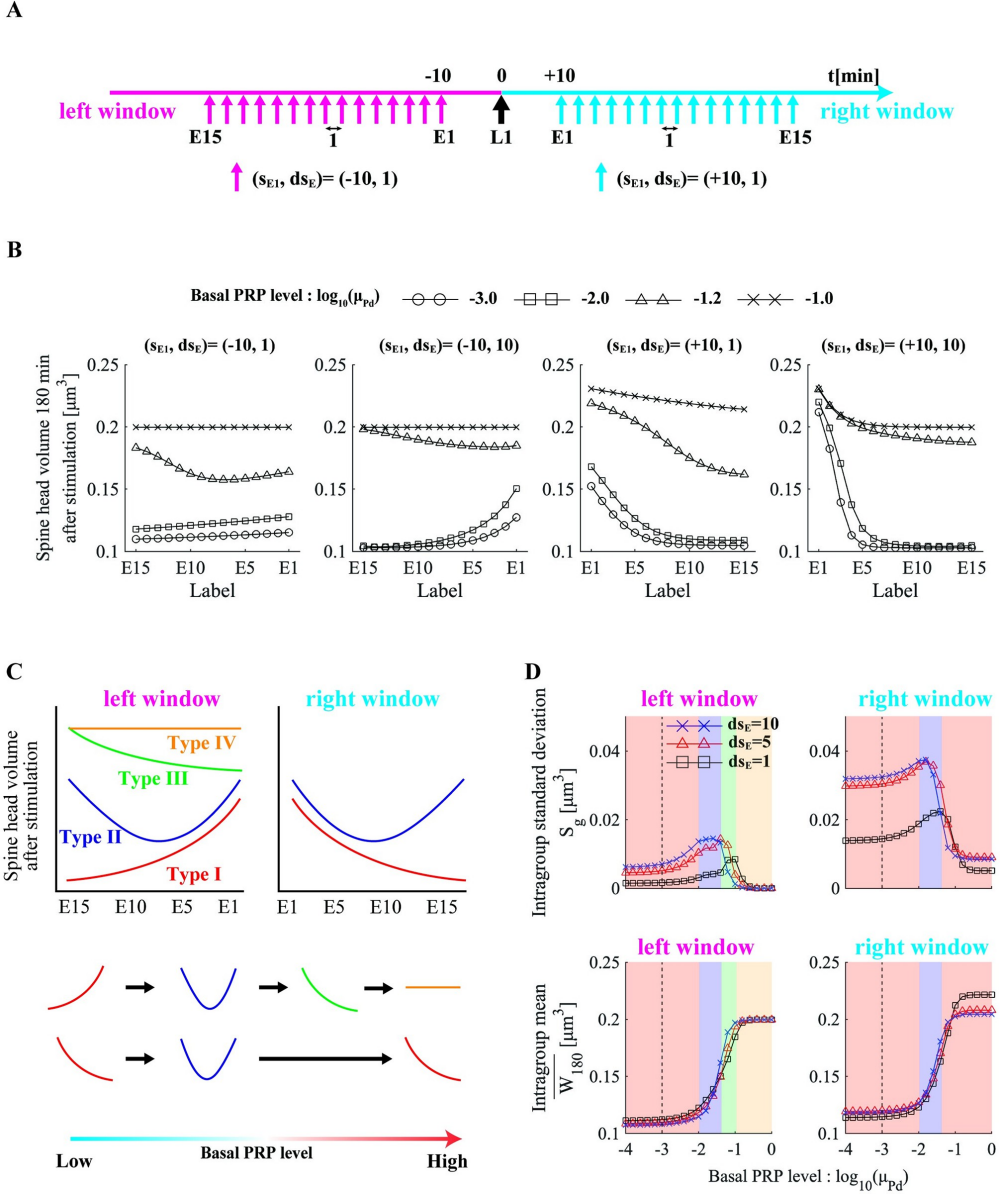
The influence of stimulation timing on late-associativity. The schematic diagram of our simulation experiment II. The horizontal axis denotes time (min). The black arrow at 0 min denotes the start of the l-LTP–inducing stimulation protocol at synapse L1. Other arrows indicate the stimuli in the e-LTP–inducing stimulation protocol at synapses E1–E15. Stimulus patterns are characterized by *s*_*E1*_ and *ds*_*E*_. *s*_*E1*_ denotes the stimulation timing at synapse E1. We defined stimulus patterns in which all e-LTP–inducing stimulation protocols were conducted before the l-LTP–inducing stimulation protocol as the left window (*s*_*E1*_ < 0) and the patterns in which the e-LTP–inducing stimulation protocols were conducted after the l-LTP–inducing stimulation protocol as the right window (*s*_*E1*_ > 0). *ds*_*E*_ is the stimulus interval. (B) Spine head volumes of synapses E1–E15 180 min after stimulation with different stimulus patterns. Marker denotes the log-scale basal PRP level in the simulation. (C) Four types of enhancement tendencies. Types I–IV alternatively appear depending on the basal PRP level in a specific order (see S1–S6 Figs for the enhancement tendencies under all stimulus patterns). (D) Basal PRP level dependency of the intragroup mean and standard deviation. Each marker line denotes a different stimulation interval. Background color denotes the range of basal PRP levels in which each type dominantly appears. Type I is red, Type II is blue, Type III is green, and Type IV is yellow.

In experiment II, we applied asynchronous stimuli to E1–En and labeled E1, E2,…, En in order from the synapse stimulated at a timing close to t = 0. In addition, we limited the freedom of stimulus timing combinations by making the stimulation interval equal. We also distinguished between the case in which the e-LTP–inducing stimulation protocol was performed before the l-LTP–inducing stimulation protocol (left window) and that in which it was performed after the l-LTP–inducing stimulation protocol (right window). In summary, asynchronous stimulation patterns were characterized by stimulation timing to synapse E1 *s*_*E1*_ and stimulation interval *ds*_*E*_ (Fig 8A). We varied the stimulation interval in the range of *ds*_*E*_ = [1,5,10] min and the basal PRP level of $\mu_{P_{d}}$ = 1 × 10^−4^–1 × 10^0^ with +0.2 increments of the exponent.

Fig 8B presents the spine head volume 180 min after stimulation for each synapse (E1–E15) when we applied four different stimulus patterns under four basal PRP levels (see S1–S6 Figs for the results of all stimulus intervals and basal PRP levels). As presented in Fig 8B, the spine head volume tended to increase as the basal PRP level increased for all stimulus sequences. Combined with the results from S1–S6 Figs, four types of synaptic volume trends are evident (Fig 8C). Type I tends to have larger volumes at synapses E1, E2,…, E15 in that order; Type III tends to have larger volumes at synapses E15, E14,…, E1 in that order; Type II has a downward convex trend with an inflection point; and Type IV has nearly equal volumes for all synapses. Interestingly, the four types appeared alternatively in the same order, i.e., Type I → Type II → Type III → Type IV in the left window and Type I → Type II → Type IV in the right window, as the basal PRP level increased regardless of the number of competing synapses or the stimulus interval (S1–S6 Figs).

Next, we characterized four synaptic enhancement types by the intragroup mean $\bar{W_{180}}$ and intragroup standard deviation *S*_*g*_ of post-enhancement volume. The formula for calculating the intragroup standard deviation was as follows:

$$S_{g}=\sqrt{\frac{1}{N}\sum_{n=1}^{N}{\left(W_{180}^{\left[n\right]}-\bar{W_{180}}\right)}^{2}}$$ (9)

where $W_{180}^{\left[n\right]}$ denotes the spine head volume 180 min after stimulation and $\bar{W_{180}}$ denotes the average of $W_{180}^{\left[1\right]}-W_{180}^{\left[N\right]}$. Note that $\bar{W_{180}}$ is different for simulations with different stimulus patterns. Fig 8D presents the basal PRP level dependence of the intragroup mean $\bar{W_{180}}$ and the intragroup standard deviation *S*_*g*_. In addition, the range of basal PRP levels in which the four types of synaptic enhancement tendencies appear predominantly is presented by the corresponding background color. As presented in Fig 8D, the basal PRP level dependence of the intragroup mean $\bar{W_{180}}$ displayed a sigmoid-type change, and it was almost independent of the stimulation interval *ds*_*E*_. Intragroup standard deviation is relatively constant at the basal PRP levels where type I and type IV appear and lower for type IV than for type I. The intragroup standard deviations and intragroup standard means change abruptly in the range of PRPs in which type II and type III appears, indicating that the relationship between large and small spine head volumes changes violently (see also S1–S6 Figs). In addition, the intragroup standard deviation tended to increase as the stimulation interval increased. The intragroup standard deviation tended to be larger in the right window than that in the left window.

## Discussion

Since Frey et al. proposed the concept of STC in a classical two-pathway experiment [6], many researchers have attempted to identify the molecular identity of synaptic tags and PRPs. However, the molecular mechanisms surrounding STC have proven to be highly complex. The functions of synaptic tags include interactions with multiple molecules, such as CaMKII and actin, and thus, the identity of synaptic tags should be described as a state rather than as a molecule [30]. PRPs contain multiple actin-binding and scaffolding proteins, and their stepwise roles are being increasingly clarified [32,39]. Despite the complexity of the molecular dynamics of STC, various phenomenological predictions based on STC have been made using simple computational models. For example, Clopath et al. successfully reproduced the qualitative behavior and cross-tagging of LTP and long-term depression (LTD) by incorporating state variables while aggregating synaptic tags and PRPs into a single variable [35]. Based on a state transition model, Barett et al. proposed a transition model between multiple stable states, including LTP and LTD [36]. Smolen et al. clarified the roles of specific molecular species in LTP and LTD by detailing the molecular mechanisms of the ERK pathway [37]. Our model reproduces STC, late-associativity, and the asymmetric temporal window of STC with a simple structure that aggregates synaptic tags and PRPs into a single variable. Thus, our model does not focus on a specific molecular cascade as in a study by Smolen et al., but it is classified as a phenomenological model as in studies by Clopath and Barett et al. In comparison with previous phenomenological models, the features of our model can be summarized as follows. (1) By considering both *de novo* synthesized PRPs and the basal PRP level in the dendritic compartment, our model reproduced the experimental results of STC under both protein-depleted and protein-rich conditions. (2) We simulated the competitive capture of PRPs across multiple synapses. In this study, we used a simple model to predict the effects of competitive PRP capture on synaptic clustering. In this section, we discuss our experimental results, model limitations, and model prospects.

### Associations of events vary based on the basic PRPs levels

Late-associativity is a concept that describes the phenomenon in which e-LTP transitions to l-LTP using a shared pool of PRPs [7]. Late-associativity is mediated by STC and is believed to occur under the following conditions: (i) e-LTP–induced synapse E1 and l-LTP–induced synapse L1 are spatially close and (ii) the timing of stimulation of E1 and L1 is temporally close [12]. Late-associativity occurs in a limited spatiotemporal window because novel protein synthesis is initiated around the stimulation site at the timing of stimulus application and late-associativity uses these *de novo* proteins. Therefore, late-associativity can aggregate signals from presynaptic neurons activated by near-timely events into spatially localized postsynaptic populations (synapse clusters). Synaptic clusters are considered helpful for encoding, saving, and retrieving memory [9]. However, some studies questioned the need for novel protein synthesis in late-associativity, and this controversy may be attributable to the different basal levels of PRPs in experimental slices [21,22]. By varying the basal PRP level in the dendritic compartment, we confirmed that the asymmetric temporal window of STC is more pronounced when the amount of PRPs in the compartment is within a specific range (Fig 4B).

What are the physiological implications of the STC temporal window and basal PRP levels? As mentioned previously, late-associativity via STC associates strong events (corresponding to the l-LTP–inducing stimulation protocol in our model) with weak events (corresponding to the e-LTP–inducing stimulation protocol in our model) based on temporal proximity. Thus, events occurring at close temporal timings are integrated into the same cluster, and the associations are strengthened. This strategy may support effective memory consolidation mechanisms in contextual memory, such as fear conditioning [40]. The asymmetry of the STC temporal window also indicates that the temporal proximity constraints of weak events preceding strong events are relatively loose, and events considered beneficial for predicting strong events may be associated even if they are temporally distant. Conversely, the temporal proximity constraints for weak events that follow strong events are tight, but weak events are strongly associated with the strong event. Thus, weak events following strong events may be associated even if they are trivial.

Interestingly, our results illustrated that with increasing basal PRP levels, the proximity constraint on the occurrence timing of weak and strong events was relaxed, weighting flattened, and eventually, clustering occurred regardless of the occurrence timing of the two events. In other words, for two events to be associated with appropriate weights based on the proximity of the timing of their occurrence, the basal PRP level must be restrained within an appropriate range. Factors that can change the basal PRP level include the presentation of novelty attributable to environmental changes as presented in prior behavioral experiments [41,42]. Circadian rhythms and the physiological states of sleep and wakefulness also alter the protein levels of neurons [43–45]. Our results suggest that changes in basal PRP levels underlie desynchronization from the circadian clock [46] and changes in memory performance dependent on the time of day and external environment [47,48].

### Competitive capture of PRPs modulates the strength of association via late-associativity

We performed experiments in which multiple synapses were subjected to asynchronous stimulation sequences (Fig 8A) and found that the relationship between the magnitude of the post-enhancement volume of each synapse varied depending on the basal PRP level, number of competing synapses, and stimulation interval. Furthermore, four types of post-enhancement volume relationships appeared alternately according to the basal PRP level (Fig 8C). Our results suggested that physiological states and the patterns of event generation are considered in the process by which responses to events are preserved as physical traces. In this section, we first discussed the reasons for the emergence of the four types. Then, we discussed the implications of each synaptic enhancement tendency for event associations.

What are the principles underlying the observed types I–IV enhancement trend? First, it should be noted that the pool of shared PRPs used in late-associativity is the sum of the basal PRP level and newly synthesized PRPs. When the basal PRP level is low, late-associativity strongly depends on *de novo* PRPs; thus, the effect of the temporal window is accentuated (type I). When the basal PRP level is increased to a certain degree, l-LTP is sufficiently induced by weak stimuli to induce synaptic tag expression (Fig 4). Thus, even if the timing of the e-LTP–inducing stimulation protocol is distant from that of the l-LTP–inducing stimulation protocol, the spine head volume is enhanced. However, when multiple synapses are stimulated with the e-LTP–inducing stimulation protocol, the number of PRPs available for capture decreases with synapse stimulated later because of the use of shared PRPs by the preceding stimulated synapses. Alternatively, the *de novo* PRPs triggered by the l-LTP–inducing stimulation protocol are available for potentiation. Thus, types II and III can appear depending on the ratio of basal PRPs to newly synthesized PRPs. An extremely high basal PRPs level results in the distribution of a sufficient amount of PRPs to all stimulated synapses regardless of the presence or absence of newly synthesized PRPs. As shown in Fig 8D, the intragroup means saturated at high values, and the intragroup standard deviation was low at high basal PRP levels. These results indicate that the differences in post-enhancement volume are small. Type IV appeared in the left window, and flattened type I appeared in the right window (Figs 8C and S1–S6). As discussed above, the types I–IV enhancement trends likely appear alternatively depending on whether newly synthesized or basal PRPs are used dominantly for enhancement.

**Fig 9:**
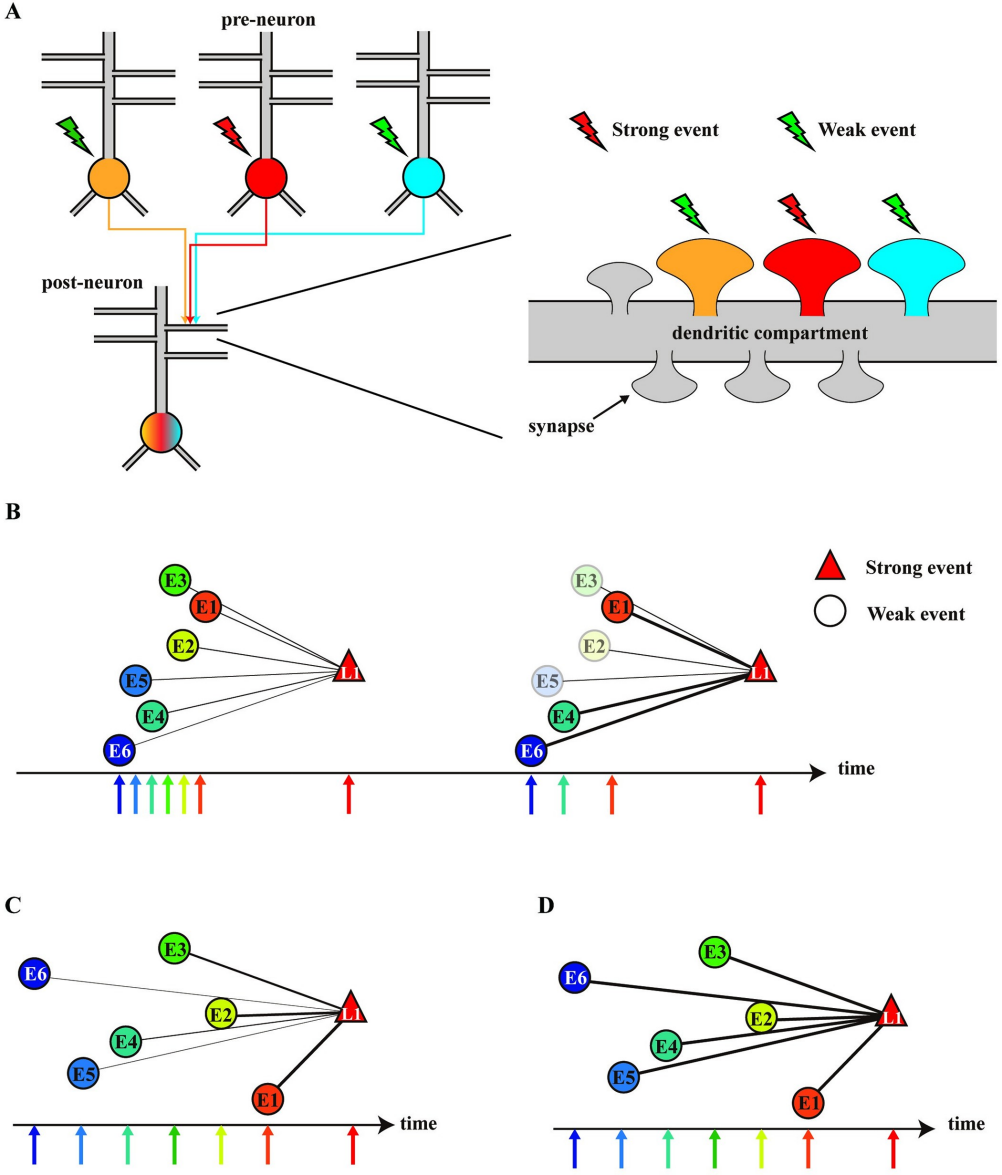
Schematic diagram of the synaptic coupling rules with competing PRP captures. (A) Illustration of synapse clustering. Each of the three preneurons is activated in response to a different event. These preneurons form spatially restricted synaptic clusters on the dendrites of the post-neuron, and the post-neuron contributes to the association of the three events. (B) If the stimulus interval is short, then each weak event is moderately associated with a strong event. In this case, the weak event, which occurs close to the strong event again, is strongly associated. (C) When the stimulus interval is long, weak events close to the strong event are strongly associated with a strong event, whereas those that occur with a different timing are weakly associated with the strong event. Thus, there is a contrast in the strength of the association. (D) When the basal PRP level is high, all synapses activated by events are strongly enhanced regardless of the timing. Thus, strong and weak events are over-associated regardless of the timing of their occurrence. In (B)-(D), line width denotes the strength of associativity.

On the other hand, Type I-IV denotes the tendency of each synapse to strengthen during the formation of synaptic clusters. Synaptic clusters make memory associations through selective strengthening connections between multiple neurons (Fig 9A) [9]. Therefore, types I–IV may have different physiological significance in memory associations. The advantages of type I for information associations are generally similar to those of late-associativity described in the previous section. Type I indicates a mechanism for associating events based on the proximity of the occurrence timing. Within the range of basal PRP levels at which type I events occur, the intragroup standard deviation increased as the stimulus interval increased in both the left and right windows (Fig 8D). These data suggest that if there is a distinct difference in the timing of weak events, weak events occurring with a similar timing as strong events will be strongly enhanced, and those occurring with a different timing will be weakly enhanced (Fig 9C). Conversely, if no distinct differences exist, the supply of PRPs is equal, and many events will be moderately enhanced. In this case, only weak event associations that occur in tandem with strong events again in the future will be relatively strengthened (Fig 9B). Second, type IV is an enhancement tendency that appears under high basal PRP levels, and there are no temporal proximity constraints on event associations, implying that all events that occur are potentiated (Fig 9D). Thus, excessive synaptic integration into synaptic clusters and reduced spatial segregation may occur. Such a condition predicts the intrusion of remote associations observed in neurological disorders such as schizophrenia [49]. Finally, the large fluctuations in intragroup mean and intragroup standard deviations during the emergence of types II and III suggest these types are volatile enhancement trends. Presumably, types II and III are intermediate states between types I and IV, a process in which the temporal proximity constraint is lost in late-associativity. Thus, types II and III may be anomalous states of information association.

In summary, our results illustrated that differences in the timing of stimulation to each synapse are reflected in the differences in the post-enhancement spine head volume. Interestingly, when the basal PRP level is in the appropriate range, weak event onset’s sparseness affects the spine head volume enhancement (Fig 9B and 9C). Our results suggest that during the formation of synaptic clusters, the selection criteria for synapses to be integrated into a cluster depend on the pattern of event onset and basal PRP levels.

### Limitations and prospects of the model

#### Spatiotemporal distribution of PRPs

Our model assumes that the distribution of PRPs within the 10-μm dendritic compartment is uniform. However, it has been suggested that the distribution of PRPs and their synthetic source, namely mRNA, varies with distance from the cell body, although the effect may be slight at the 10-μm scale [50,51]. Therefore, when extending our model spatially, it would be interesting to assess the impact of the nonuniform distribution of PRPs on our experimental results. Alternatively, stimulation repositions polyribosomes at the base of large spines, and the distribution and maturity of spines could bias local synaptic potentiation [26]. It has also been suggested that the structure of microdomains, such as endoplasmic reticula and endosomes, change depending on the dendritic maturity, resulting in changes in the protein distribution [24]. Incorporating age-dependent changes in the protein distribution into models and tracking changes in synaptic cluster formation will be significant in analyzing age-related changes in memory performance.

#### Single-neuron model with multiple dendrites

Spines and dendrites are computational units of signal integration with different nonlinearities [52,53]. Local signal integration by synaptic clusters has also been suggested to improve the memory capacity of single neurons and to be important in memory allocation [9,10]. Although our model predicts that the basal level of PRPs in local compartments and the interval between event occurrences affect the formation of local synaptic clusters, it would be interesting to analyze the extent to which local modulations affect the computational results for the whole neuron. As mentioned in the previous section, the basal PRP level in dendrites depends on their distance from the soma [50,51]. The ratio of locally synthesized to soma-derived PRPs may regulate interference between memory engrams [54]. Future work is needed to extend our model to entire neurons to analyze the interplay between local and global regulation and the role each plays in memory allocation.

#### Effect of the competitive capture of proteins on plasticity on a long time scale

The mechanism by which the functional and structural properties of the enhanced spine are maintained remains controversial [55]. The proteins that comprise the spine are constantly turning over, and thus, the synapse must constantly capture proteins to maintain the l-LTP state. Alternatively, proteins might be explicitly synthesized at the enhanced spines. Some researchers believe a positive feedback loop is part of the mechanism that maintains l-LTP [56–58]. Conversely, it has been reported that l-LTP is not lost when protein synthesis inhibitors are applied to spines more than 4.5 h after l-LTP induction [22]. Our model assumes that the l-LTP state is maintained for a long time with or without PRPs. Thus, the volume of potentiated synapses does not decay within the simulation time (Eq (7)). Fonseca et al. reported that l-LTP is lost 4 h after l-LTP induction when competition occurs in the presence of protein depletion [18]. Our model cannot reproduce this phenomenon. However, our focus was on the competitive capture of PRPs, which occurs in parallel with PSD enhancement approximately 60 min after l-LTP induction [19,32,33]. Thus, the assumptions we made in our model do not affect the simulation results. In the future, once the molecular mechanism by which l-LTP is maintained is clarified, we will be able to assess the impact of the competitive capture of proteins on later plasticity stages and discuss the dynamics of plasticity on a longer time scale.

#### Can the model explain the various types of plasticities?

Our model can simulate e-LTP and l-LTP, but by extension, e-LTD and l-LTD can also be implemented. The simplest strategy is to introduce an LTD tag, as employed in previous phenomenological models [35,37]. Because LTP and LTD utilize a shared pool of PRPs [7], two types of plasticity can be distinguished by providing two different tags. If LTP and LTD arise competitively, this may explain the diverse patterns seen in heterosynaptic plasticity [59]. However, the relationship between LTP and LTD is inherently more complex, and the molecular mechanisms common to LTP and LTD need to be clarified. At least, LTP and LTD are regulated by several common GTPases [60]. Models focusing on AMPAR diffusion and transport and considering dendrite and spine morphology suggest that a change in the balance between the endocytosis and exocytosis of AMPARs distinguishes LTP and LTD [61,62]. In addition, the competitive capture of AMPARs could lead to heterosynaptic plasticity [63,64]. Alternatively, Kirchner et al. used a neurotrophin model to predict heterosynaptic plasticity based on distance-dependent competition from the soma and timing-dependent cooperation of synaptic activity [65]. On the contrary, apart from competitiveness, Oh et al. reported that heterosynaptic plasticity could occur in a space as narrow as 4 μm because of the spatial diffusion of calcineurin [66]. Such negative regulatory signals may coexist with the competitive mechanisms, including competitive PRP capture, which was the focus of this analysis. Notably, late-associativity can occur over a spatial range of up to 70 μm along dendrites [12]. If the effects of the competitive capture of PRPs occur over an ample space, heterosynaptic plasticity could be the product of a combination of factors at different spatial scales. Integrating the effects of the protein distribution across dendrites on plasticity into our model, as discussed by Kastellakis [54] and Fonkeu [51], could allow us to analyze the impact of different spatiotemporal scale mechanisms for PRP capture on memory regulation.

## Supporting information

S1 Fig The influence of stimulation timing on late-associativity with (*N*, *s*_*E1*_) = (5, -10). The horizontal axis denotes the labels of synapses. The vertical axis denotes the spine head volume of E1-En 180 min after the e-LTP–inducing stimulation protocol. The basal PRP level (log_10_ scale) is shown at the top of each panel. Each marker line indicates different stimulus intervals.
(TIF)

S2 Fig The influence of stimulation timing on late-associativity with (*N*, *s*_*E1*_) = (10, -10). The figure shows the same contents as S1 Fig. However, the number of competing synapses *N* and the stimulation timing at synapse E1 *s*_*E1*_ are different.
(TIF)

S3 Fig The influence of stimulation timing on late-associativity with (*N*, *s*_*E1*_) = (15, -10). The figure shows the same contents as S1 Fig. However, the number of competing synapses *N* and the stimulation timing at synapse E1 *s*_*E1*_ are different.
(TIF)

S4 Fig The influence of stimulation timing on late-associativity with (*N*, *s*_*E1*_) = (5, +10). The figure shows the same contents as S1 Fig. However, the number of competing synapses *N* and the stimulation timing at synapse E1 *s*_*E1*_ are different.
(TIF)

S5 Fig The influence of stimulation timing on late-associativity with (*N*, *s*_*E1*_) = (10, +10). The figure shows the same contents as S1 Fig. However, the number of competing synapses *N* and the stimulation timing at synapse E1 *s*_*E1*_ are different.
(TIF)

S6 Fig The influence of stimulation timing on late-associativity with (*N*, *s*_*E1*_) = (15, +10). The figure shows the same contents as S1 Fig. However, the number of competing synapses *N* and the stimulation timing at synapse E1 *s*_*E1*_ are different.
(TIF)
