# Supplementary figures and images for: A computational model to explore how temporal stimulation patterns affect synapse plasticity
Source: PLoS One. 2022 Sep 23;17(9):e0275059. doi: 10.1371/journal.pone.0275059 (PMC9506666; doi:10.1371/journal.pone.0275059)

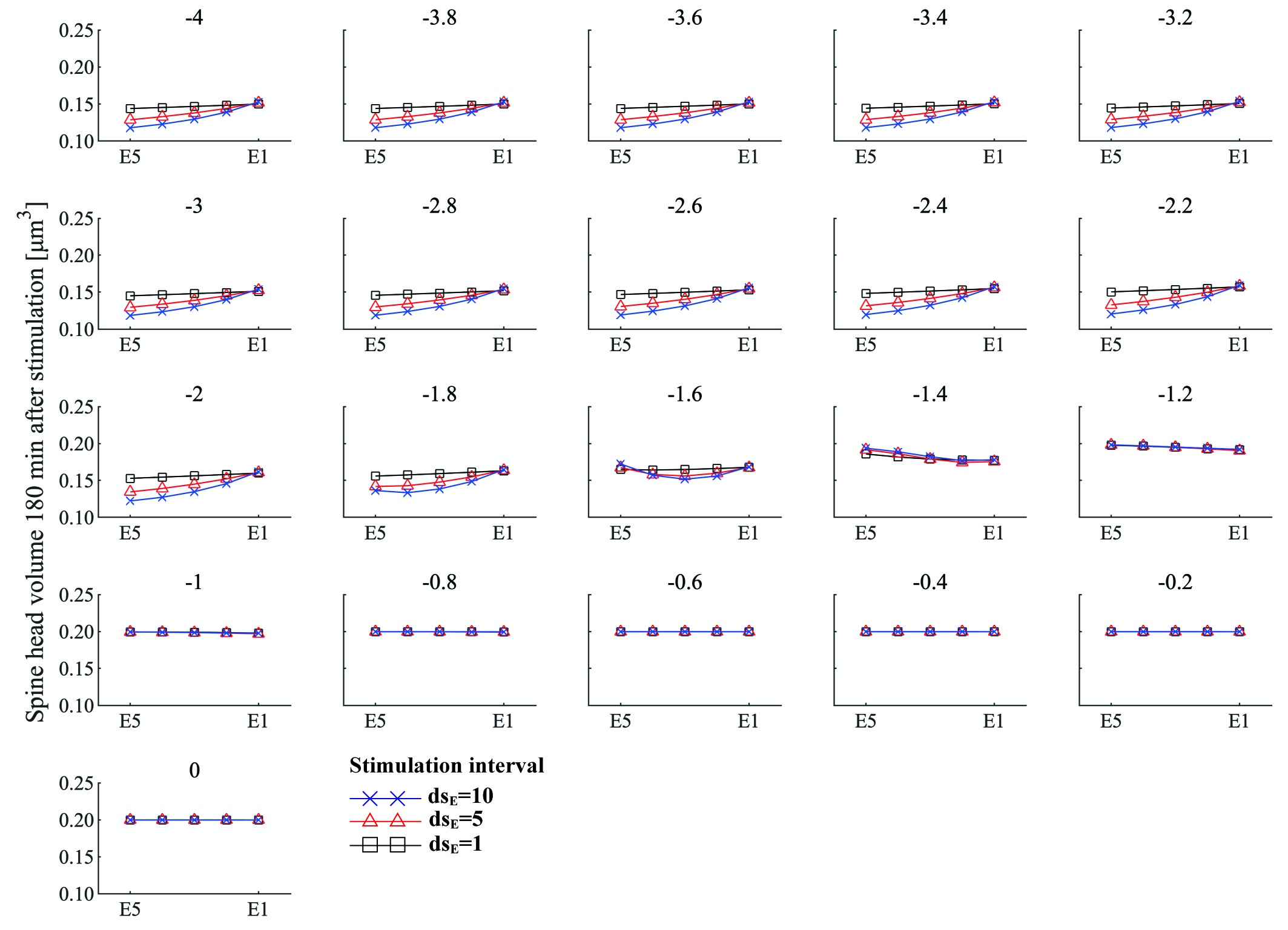

Supplement: S1 Fig — The horizontal axis denotes the labels of synapses. The vertical axis denotes the spine head volume of E1-En 180 min after the e-LTP–inducing stimulation protocol. The basal PRP level (log10 scale) is shown at the top of each panel. Each marker line indicates different stimulus intervals. (TIF) [file pone.0275059.s001.tif]

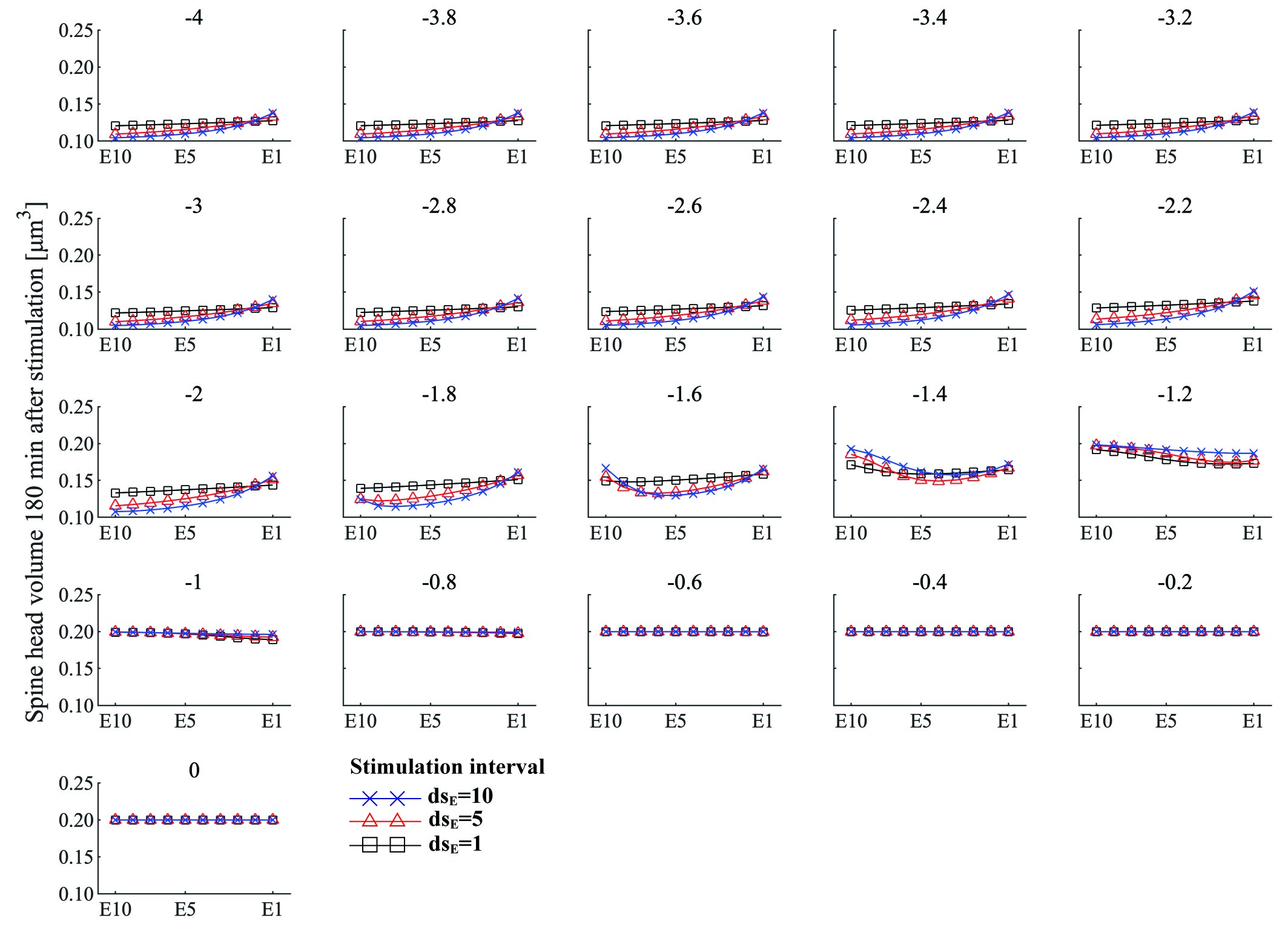

Supplement: S2 Fig — The figure shows the same contents as S1 Fig. However, the number of competing synapses N and the stimulation timing at synapse E1 sE1 are different. (TIF) [file pone.0275059.s002.tif]

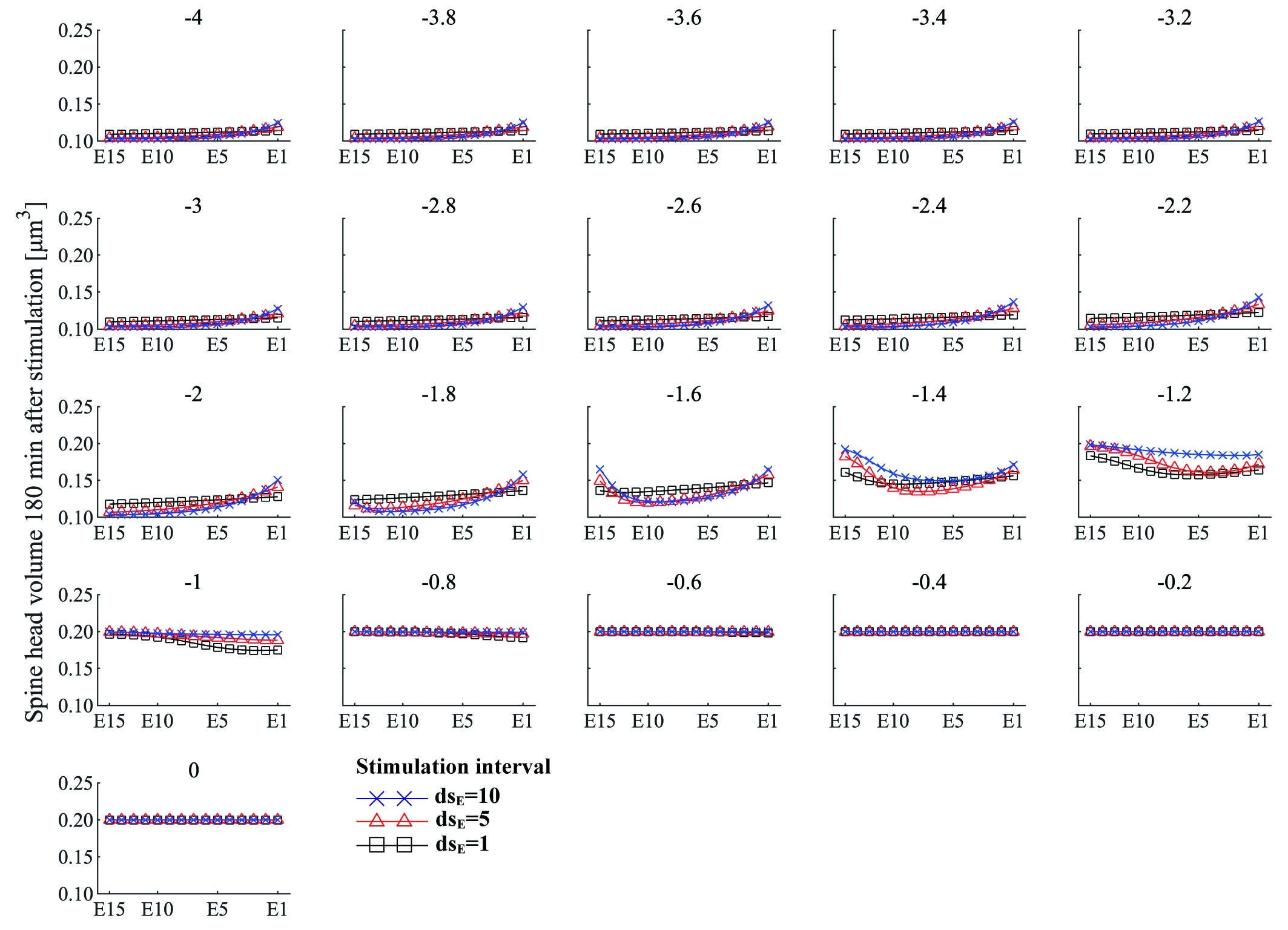

Supplement: S3 Fig — The figure shows the same contents as S1 Fig. However, the number of competing synapses N and the stimulation timing at synapse E1 sE1 are different. (TIF) [file pone.0275059.s003.tif]

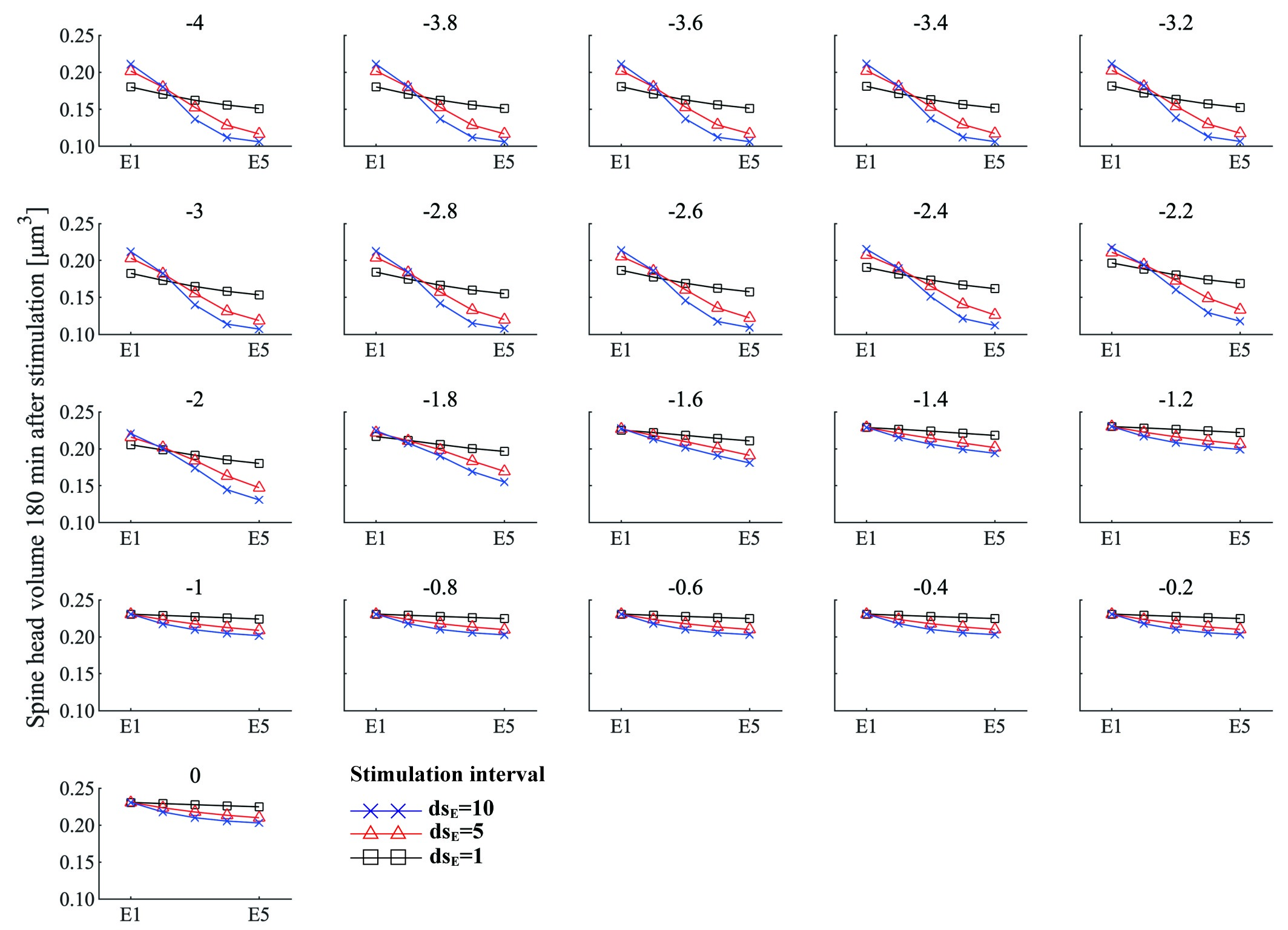

Supplement: S4 Fig — The figure shows the same contents as S1 Fig. However, the number of competing synapses N and the stimulation timing at synapse E1 sE1 are different. (TIF) [file pone.0275059.s004.tif]

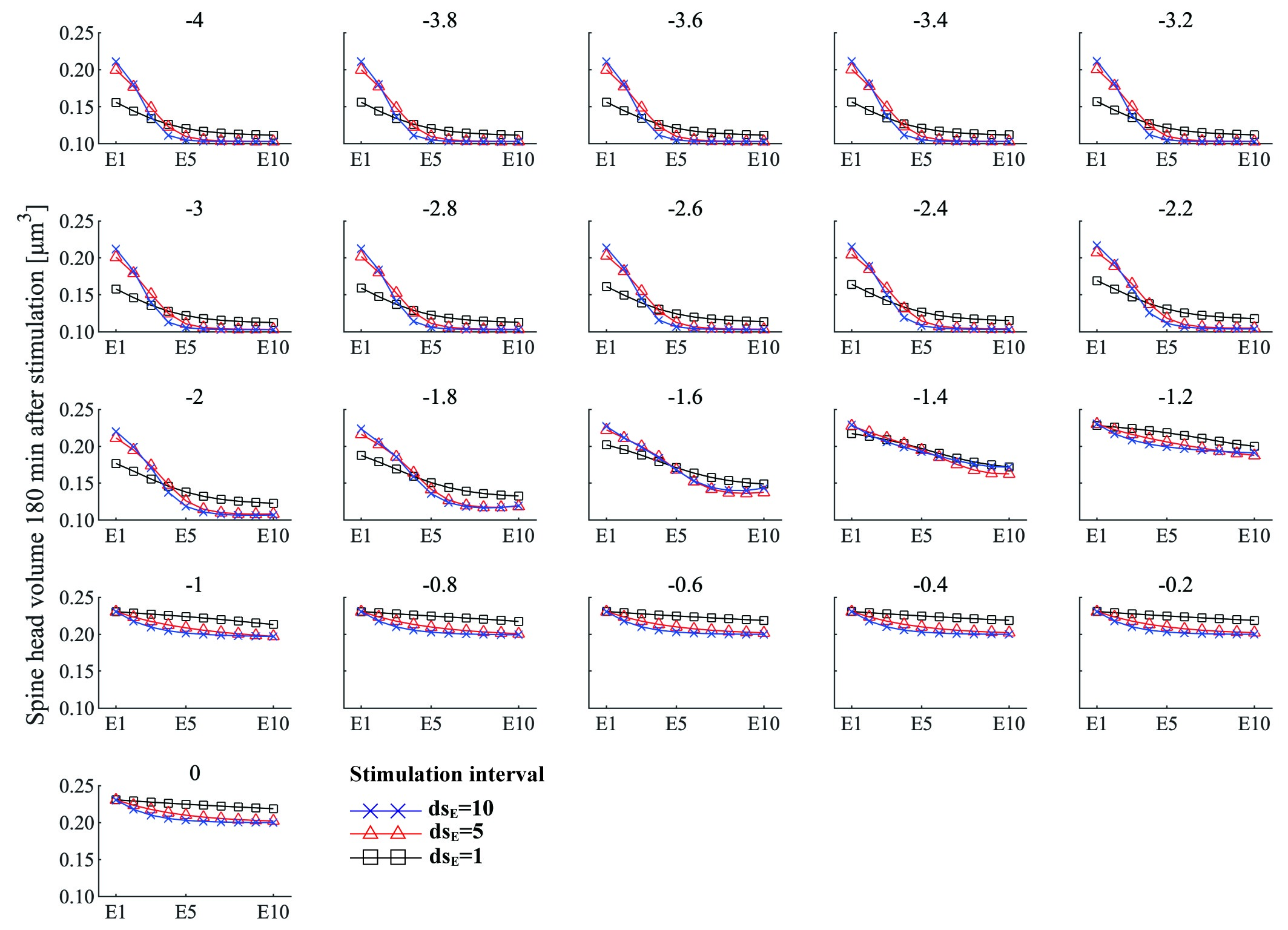

Supplement: S5 Fig — The figure shows the same contents as S1 Fig. However, the number of competing synapses N and the stimulation timing at synapse E1 sE1 are different. (TIF) [file pone.0275059.s005.tif]

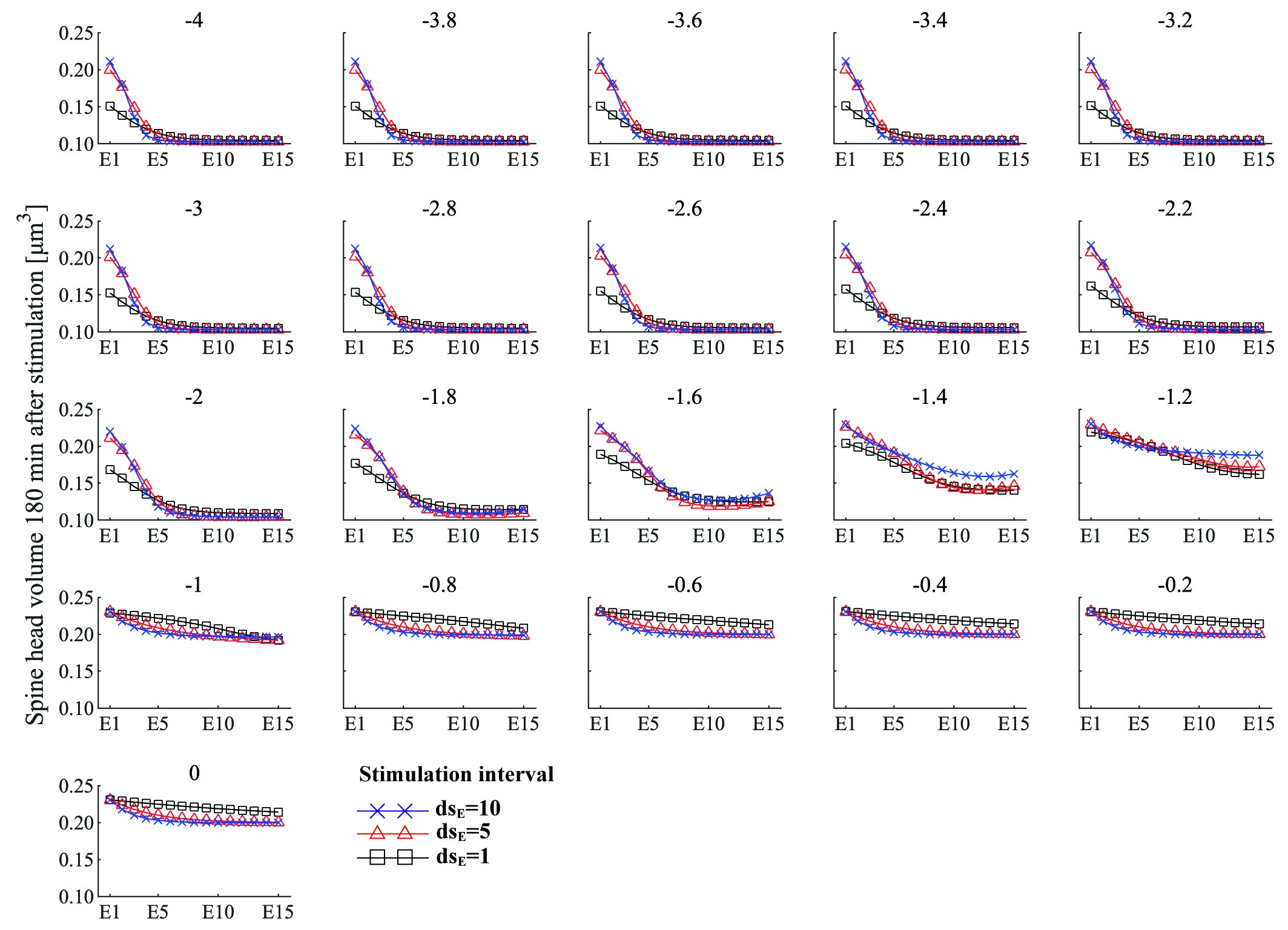

Supplement: S6 Fig — The figure shows the same contents as S1 Fig. However, the number of competing synapses N and the stimulation timing at synapse E1 sE1 are different. (TIF) [file pone.0275059.s006.tif]
